# Volatiles of the entomopathogenic fungus, *Metarhizium brunneum*, attract and kill plant parasitic nematodes

**DOI:** 10.1016/j.biocontrol.2020.104472

**Published:** 2021-01

**Authors:** Salim Khoja, Khalifa M. Eltayef, Ian Baxter, Arben Myrta, James C. Bull, Tariq Butt

**Affiliations:** a Department of Biosciences, Swansea University, Singleton Park, SA2 8PP. Swansea, UK; b Certis Europe BV, Stadsplateau 16, 3521 AZ Utrecht, the Netherlands

**Keywords:** Plant parasitic nematodes, Fungal volatiles, *Metarhizium*, Repellents, Attractants

## Abstract

Root knot nematodes (RKNs) cause significant crop losses. Although RKNs and entomopathogenic fungi, such as *Metarhizium brunneum*, are associated with plant roots, very little is known about the interactions between these two organisms. This study showed that conidia and VOCs of *Me. brunneum* influenced the behaviour of *M. hapla*. The response was dependent on the fungal strain, VOC, concentration of both VOC and conidia, and time. Tomatoes planted in soil treated with the highest doses of conidia usually had a higher number of nematodes than untreated control plants. This was particularly obvious for *Me. brunneum* strain ARSEF 4556, 7 and 14-days post-treatment. The VOCs, 1-octen-3-ol and 3-octanone, lured *M. hapla* to plants when used at low doses and repelled them at high doses. In Petri dish assays. the VOCs 1-octen-3-ol and 3-octanone, caused 100% mortality of *M. hapla* at the highest dose tested (20 µl). Very few live *M. hapla* were recovered from soil treated with the VOC 1-octen-3-ol, especially at the highest doses tested.

## Highlights

• *M. hapla* J2s are attracted to plant roots treated with *Me. brunneum* conidia.
• J2s are attracted to roots treated with low doses of 1-octen-3-ol and 3-octanone.
• High doses of 1-octen-3-ol and 3-octanone kill or repel J2 *M. hapla*.
• The VOCs 1-octen-3-ol and 3-octanone show promise as nematicides.

## Graphical abstract

**Figure:**
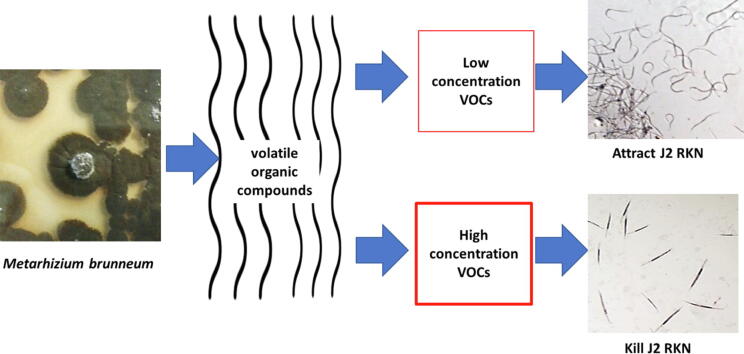

## 1 Introduction

Plant parasitic nematodes (PPN) cause 12–14% global crop losses annually (Nicol et al., 2011, Singh et al., 2015). They cause damage directly through feeding and through transmission of plant diseases (Back et al., 2002, Siddiqui et al., 2014). Some PPN, such as *Meloidogyne chitwoodi* Golden, O'Bannon, Santo & Finley, can continue to cause damage to produce even when in cold storage, reducing the marketability of the product (Ingham et al., 2000). Root knot nematodes (RKN) belonging to the genus *Meloidogyne* rank among the most destructive of PPN, they account for 5% of global crop losses (Sasser and Carter, 1985). The genus includes over 90 species with some having several races. There are several economically important species including *Meloidogyne incognita* (Kofold & White) Chitwood, *M. hapla*, *M. javanica* (Treub) Chitwood, *M. arenaria* (Neal) Chitwood, *M. chitwoodi* and *M. graminicola* Golden & Birchfield*.*

Control of PPN poses a serious challenge. The rotation and resistance of crop varieties are complementary strategies to control PPN, but their effectiveness is limited (Jones et al., 2013). Many chemical control agents have been withdrawn or restricted in their use due to the risks they pose to human health and the environment. Several microbial biological control agents (BCAs) have been developed for PPN control including *Pasteuria penetrans* Sayre and Starr*, Pochonia chlamydosporia* (Goddard) Zare and Gams and *Purpureocilium lilacinum* (Thom) Luangsa-ard, Hou- braken, Hywel-Jones and Samson (Abd-Elgawad and Askary, 2018, Li et al., 2015, Stirling, 2018). The drawback with microbial BCAs is that they are usually slow acting, target specific developmental stages and are often restricted to specific PPN species (Bontempo et al., 2014, Dahlin et al., 2019, Viggiano et al., 2014). Recent studies show that several rhizosphere competent bacteria and fungi produce volatile organic compounds (VOCs) with nematicidal properties (Bui et al., 2019, Xu et al., 2015, Yang et al., 2012, Zhai et al., 2018). The VOCs vary in chemistry and potency with some being more potent nematicides than some commercial chemical pesticides (Xu et al., 2015). Entomopathogenic fungi (EPF) belonging to the genera *Metarhizium* and *Beauveria* also produce VOCs which influence insect behaviour (Butt et al., 2016). Recently, two *Metarhizium*-derived VOCs, 1-octen-3-ol and 3-octanone, were shown to repel or kill molluscs (Khoja et al., 2019). Both compounds occur naturally; they are produced by many fungi and plant species and are used in the food flavouring and perfume industries (Lee et al., 2005, Ravi et al., 2019, Socaci et al., 2009, Torregiani et al., 2017). They also appear to play a major role in fungus-arthropod interactions, particularly as signalling compounds (Holighaus and Rohlfs, 2019). VOCs offer much potential as fumigants, to eradicate free-living stages of PPN. Due to their ephemeral nature, 1-octen-3-ol and 3-octanone, have many advantages over conventional fumigants, which have been phased out or are restricted in use. The aim of the current study was to investigate the potential of 1-octen-3-ol and 3-octanone as nematicides and nematode repellents.

## 2 Materials and methods

### 2.1 Maintenance of *Meloidogyne hapla*

A colony of *M. hapla* was maintained on tomato (*Solanum lycopersicum* L. variety Moneymaker) grown in a glasshouse at 23 ± 3 °C. Roots of 6-month old infested plants were rinsed free of soil with tap water and the nematodes harvested using a modified version of the Baermann funnel method (Barker et al., 1985, Southey, 1986). Assays were performed using second-stage juveniles (J2), the first moult having occurred within the egg. Newly hatched J2 are free living and do not feed until they locate a host root. Healthy J2s were used within 2 days of emergence from eggs in all assays unless indicated otherwise.

### 2.2 Maintenance of *Me. brunneum* isolates

The origin and growth of *Me. brunneum* strains V275 and ARSEF4556 on Sabouraud Dextrose Agar and broken Basmati rice is described by Ansari and Butt (Ansari and Butt, 2011). The identity of the strains was validated using molecular markers (Kortsinoglou et al., 2020). Conidia were harvested from the surface sporulating cultures on SDA or rice grain as outlined by Butt and coworkers (Ansari and Butt, 2011, Khoja et al., 2019).

### 2.3 Nematode survival in the presence of *Me. brunneum* conidia

**Fig. 1:**
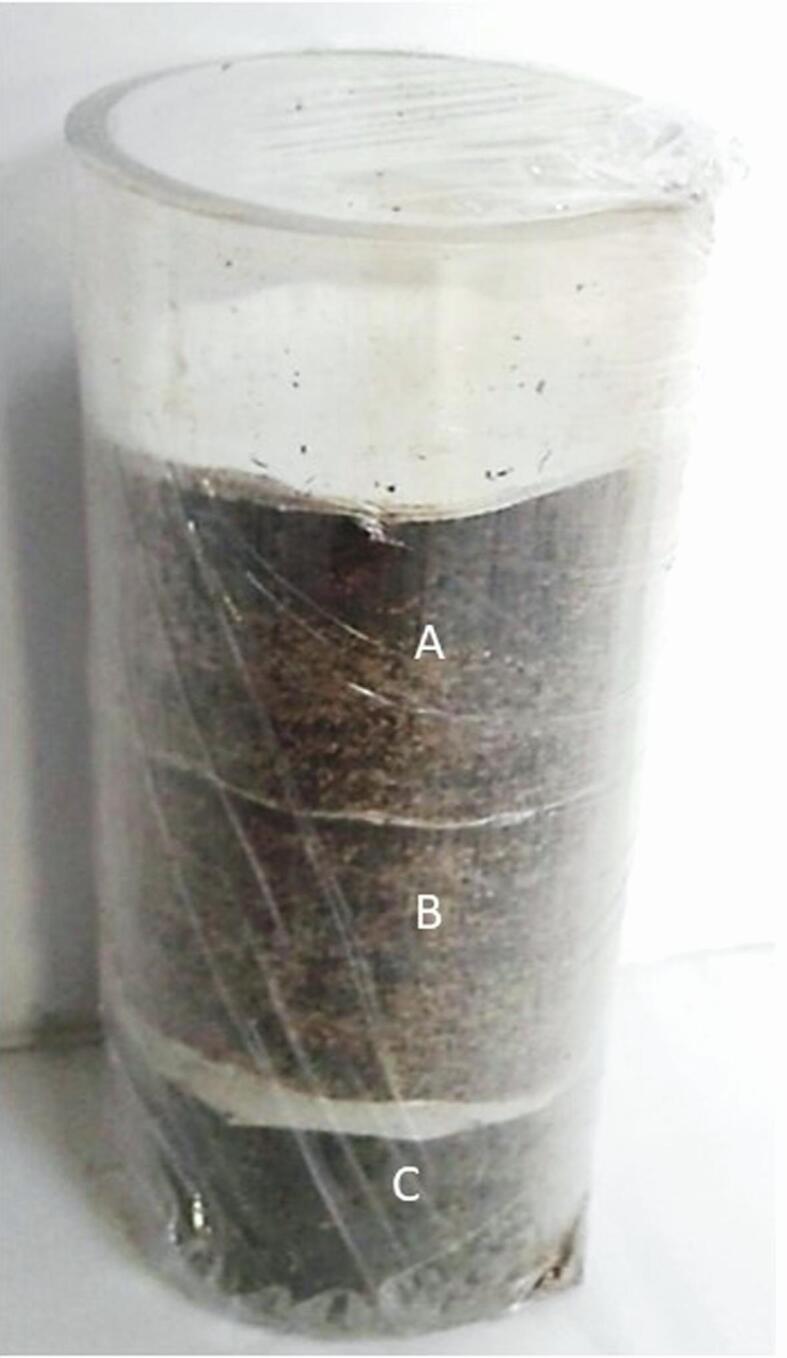
Open-ended, clear, extruded acrylic tubes (8 cm height ×2cm diameter) used to assay *M. brunneum* conidia and volatile organic compounds (VOCs). The column was filled with 21 g treated and untreated soil. The soil was divided into three 7 g layers (A: Top, B: Middle and C: Bottom) with each layer being partitioned by two layers of muslin cloth (28 mm diameter) to allow free movement of water and nematodes The nematode suspension was applied to the surface of the soil.

To determine if *Me. brunneum* affected *M. hapla* survival, three doses (1, 5, 10% w/w) of air-dried conidia of *Me. brunneum* V275 and ARSEF4556 were uniformly mixed into moist soil (Growise Organic Garden Compost). Weight wise there were approximately 1 × 10^9^ conidia per gram. Untreated soil acted as a control. In all studies the sterile soil was used unless indicated otherwise. The soil was sterilized by autoclaving at 120 °C for 30 min, Open-ended clear extruded acrylic tubes (56 ml, 8 cm height × 2 cm diameter) were filled with 21 g treated and untreated soil. The soil was divided into three 7 g layers (A: Top, B: Middle and C: Bottom) with each layer being partitioned by two layers of muslin cloth (28 mm diameter) to allow free movement of water and nematodes (Fig. 1). A 2 ml aqueous suspension of about 400 J2 *M. hapla* was applied to the soil surface. Each tube was immediately sealed at both ends with Parafilm (Caterwrap) to minimise any loss of *Me. brunneum* volatiles.

**Fig. 2:**
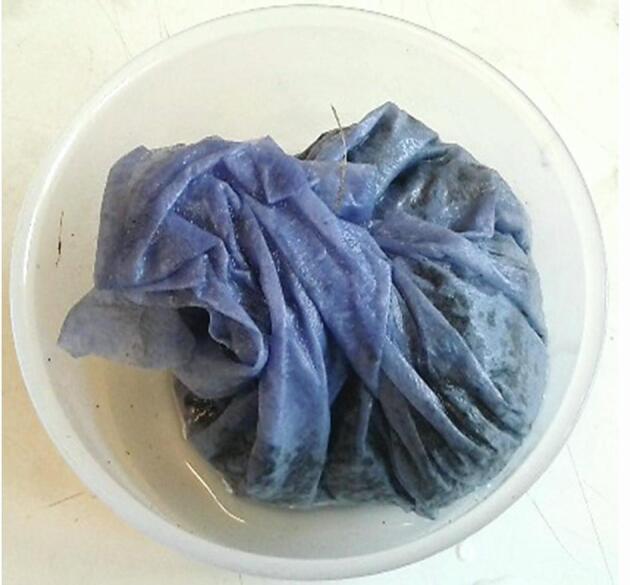
Soil wrapped in blue tissue paper was placed in a 300 ml beaker containing 20 ml water. Nematode adults and juveniles migrate from the soil through the tissue paper into the surrounding water. Samples of water could be examined in the microscope to quantify the nematodes at different time points. (For interpretation of the references to colour in this figure legend, the reader is referred to the web version of this article.)

Samples were incubated at 20 ± 3 °C in the dark. J2s were recovered 5 and 10-days post treatment. An innovative recovery method was used which entailed incubating each soil layer in blue tissue placed in a 300 ml beaker containing 20 ml sterile water (Fig. 2). Healthy J2 migrated into the surrounding water and 1 ml samples were taken on days 2, 3 and 4 post-incubation. The nematodes were immediately counted in a microscope (Kyowa Optical). There were three replicates per treatment with the whole study being conducted twice.

### 2.4 Behaviour of *M. hapla to Me. brunneum* conidia in choice study

**Fig. 3:**
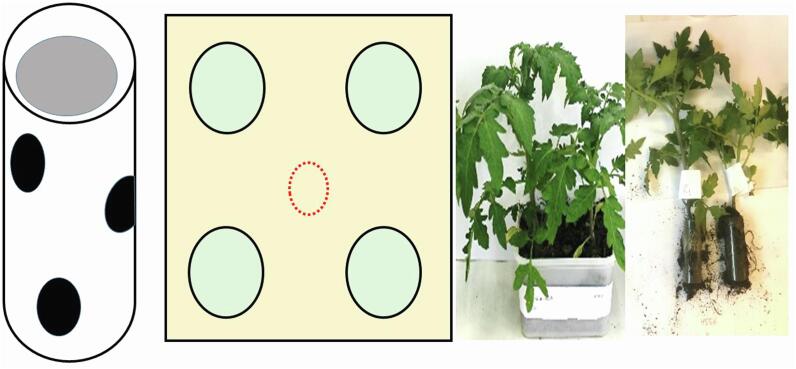
Cartoons and photographs of assay columns and arenas used for choice and no choice studies to determine the influence of *M. brunneum* conidia and VOCs on *M. hapla* behaviour. Thirty-day-old tomato plants (var Moneymaker) were transplanted into acrylic tubes (8 cm × 2 cm dia) which had several holes to allow entry of nematodes. These were planted at the corners of 2.5L plastic containers (18 × 18 × 9 cm) containing 3 L of soil. Nematodes were released at the centre (red, dashed circle) of the arena. The columns could then be retrieved to count the nematodes in each column. (For interpretation of the references to colour in this figure legend, the reader is referred to the web version of this article.)

PPN typically migrate to host plant roots. A choice study was performed to determine if *Me. brunneum* conidia influenced the attractiveness of tomato roots to *M. hapla*. Three doses (1%, 5% and 10% w/w) of air-dried conidia of *Me. brunneum* strains V275 and ARSEF 4556 were mixed in sterile soil as described above. Untreated soil was used as a control. Thirty-day-old tomato plants (var Moneymaker) were transplanted into 21 g soil in 56 ml clear acrylic tubes which had 6 evenly spaced 5 mm diameter holes (Fig. 3). The holes allowed entry of nematodes and diffusion of fungal volatiles into the surrounding soil. The plants were planted in 2L soil in 2.5L plastic containers (18 × 18 × 9 cm) (Fig. 3). In the choice study, 2 treated plants were planted one side of the container and *Metarhizium* free control plants planted at the opposite end (Fig. 3). The container was inoculated by applying 4 ml of an aqueous suspension of about 800 J2 *M. hapla* at the centre of the container. Assays were conducted in a greenhouse at 23 ± 3 °C and assessments made weekly i.e. one treated and untreated plant assessed 1-week post treatment and the second set assessed 2 weeks post treatment. The photoperiod for the glasshouse studies was 10hrs light and 14hrs dark. Nematodes were recovered from the soil in the tubes and quantified as described earlier. There were three replicates per treatment and whole study repeated twice.

### 2.5 Petri dish VOC fumigation assay against *M. hapla*

**Fig. 4:**
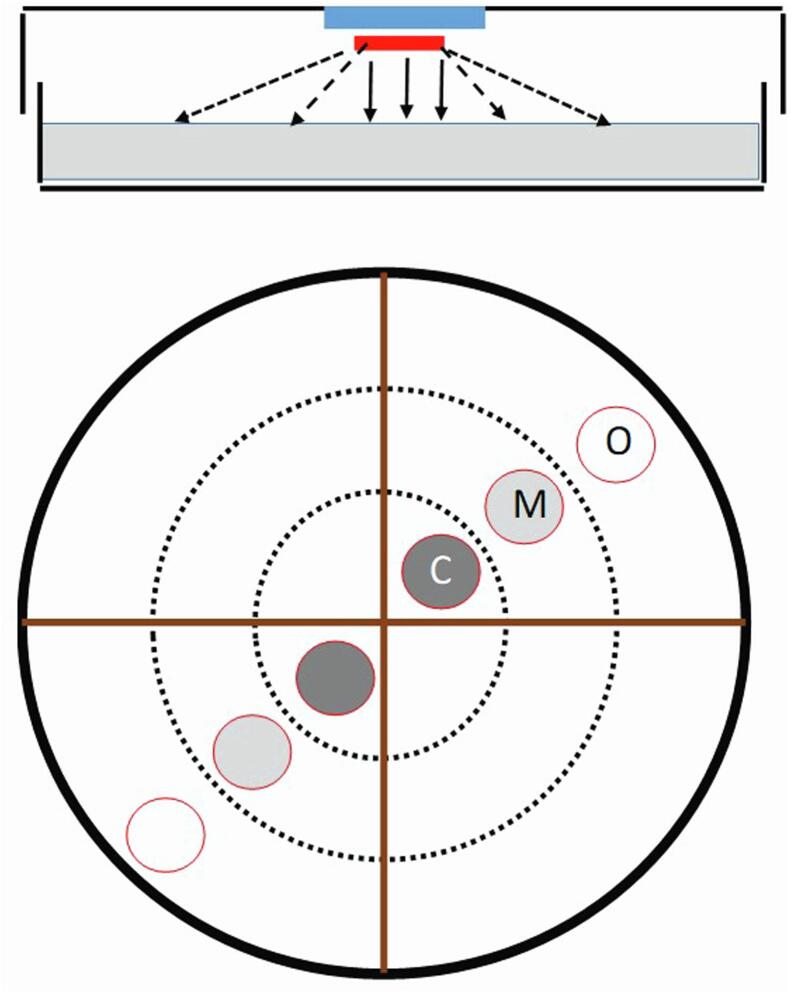
Petri dish assay to determine effect of VOC dose on nematode survival. The VOC is dispensed from filter paper attached to glass coverslip adhering to inner face of Petri dish lid (Upper figure). Nematodes spread over thin layer of water agar. Samples taken along transect from three zones representing different exposure doses (Bottom figure). Nematode mortality corresponded with VOC dose. Abbreviations: O = outer zone where there is little or no deposition of VOC; M = middle zone where there is moderate deposition of VOC; C = central zone where there was the highest deposition of VOC.

To determine if *Me. brunneum* VOCshad nematicidal activity, 1-octen-3-ol (CAS number 3391-86-4) and 3-octanone (CAS number 106–68-3) purchased from Sigma-Aldrich (>98% purity) were screened against *M. hapla* using a simple petri dish assay. Briefly, a 1 ml suspension of 150 J2 *M. hapla* was spread uniformly over the surface of 3.8% w/v water agar (Agar ash 2.0–4.5%, Sigma Aldrich). Each 9 cm diameter petri dish contained 15 ml of water agar. Nematodes were exposed to different doses (5, 10 and 20 µl) of the test compound released from 0.5 cm diameter Whatman® Grade 1 qualitative filter paper. The latter was attached to a glass coverslip (22 mm × 22 mm) attached to the centre of the petri dish lid (Fig. 4A). The petri dishes were sealed with Parafilm (Caterwrap) and incubated for 3, 6, and 24 hrs at room temperature in the dark. To determine nematode viability, a transect consisting of a 2 cm wide band was drawn across the centre of the dish within which six evenly spaced 1 cm diameter circular zones were drawn (Fig. 4B). The dead and live nematodes in these circles were counted using a stereo binocular microscope. The nematodes were classified as alive if they were mobile and possessed a sigmoidal shape but were considered dead if they were unable to move after being probed with a needle.

Petri dishes without any VOCs were used as a control. There were 9 replicates per treatment and the study was repeated twice.

### 2.6 Efficacy of formulated VOCs as a fumigant to eradicate PPN in soil

The assay was the same as for pure conidia except the conidia were replaced by a cyclodextrin granular formulation of 1-octen-3-ol (9.8% v/w) provided by Certis Europe BV. Three doses (1%, 5%, 10% w/w) of granules were evaluated. Nematode emergence was determined as outlined earlier. The average a one ml of extracts (aqueous suspension) were examined after 2, 3 and 4 days for each sample. J2 *M. hapla* were immediately counted under microscope (Kyowa Optical). There were three replicates per treatment with the whole study being conducted twice.

### 2.7 Response of *M. hapla* to VOCs in choice studies

Two assays were used to determine *M. hapla* responses to the VOCs. The first assay entailed use of a granular formulation of 1-octen-3-ol (9.8% v/w) which was mixed in sterile soil at three concentrations (1, 5, 10% w/w). The perforated 56 ml acrylic tubes were filled with treated and untreated (control) soil. The second assay entailed use of three doses (50, 100 and 200 μl) of pure liquid 1-octen-3-ol and 3-octanone dispensed from 7 mm dia Sharrow cellulose filter tips (Wilsons & Co Ltd). The filters were attached to the 56 ml perforated tube. For both assays, 30-day old tomato plants (variety Moneymaker) were transplanted into the tubes then transferred to 3L of soil in rectangular (32 × 15 × 12 cm) containers. Two treated plants were placed at one end of the container and two untreated controls at the other end. A 4 ml suspension of about 800 J2 *M. hapla* was applied using a pipette to the centre. The studies were conducted in a greenhouse at 23 ± 3 °C and 40–60% RH. The tubes were removed weekly for 2 weeks respectively and nematodes recovered from the soil taken from the tubes as described earlier. There were three replicates for the first assay and five replicates for the second assay per treatment and the whole study was repeated twice.

### 2.8 Effect of VOCs on *M. hapla* behaviour in the presence of host plant

To see if *M. hapla* J2s avoided plants treated with VOCs, a no choice study was conducted using two doses (1 and 5% w/w) of cyclodextrin granular formulations of 3-octanone and 1-octen-3ol (9.98% v/w) mixed in compost as described earlier. Treated and untreated (control) soil (21 g) was added to 56 ml perforated tubes before transplanting with 30-day old tomato plants (var. Moneymaker). Each tube was planted in sterilized soil in 0.5L plastic pots (10x8x8cm) before pipetting 4 ml aqueous suspension of about 800 J2 *M. hapla* to the soil surface. All experiments were conducted in the greenhouse at 23 ± 3 °C and 40–60% RH. Plants in tubes were uprooted 30 days later and nematodes extracted from the VOC-treated and control soils and quantified as described earlier. There were three replicates per treatment and the study was repeated twice.

### 2.9 Statistical analysis

Numbers of nematodes were modelled as over-dispersed count data using Generalised Linear Models (GLMs) with quasipoisson error structure and log link functions. Proportional mortality was estimated from quasibinomial GLMs with logit link functions, fitted to numbers of alive and dead nematodes. Since data collected at different time points was observed using different individuals, there was no issue of temporal correlation or censoring typically associated with longitudinal bioassays. All explanatory variables were initially incorporated as fully interacting, corresponding to our experimental design. Significance of terms was assessed using F-tests and non-significant terms removed. All statistical analysis was conducted using R version 3.6.1 (Team, R. Core, 2019).

## 3 Results

### 3.1 Nematode survival in the presence of *Me. brunneum* conidia

**Fig. 5:**
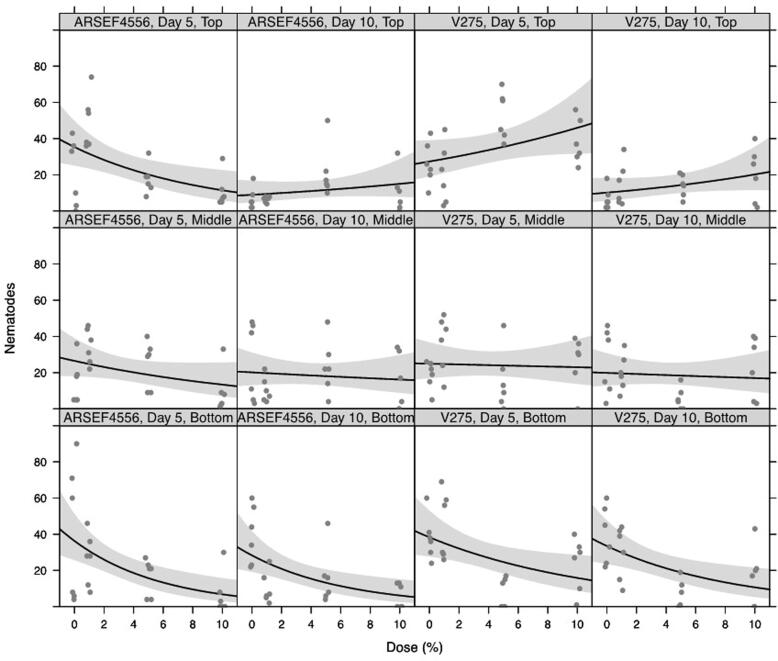
Dose-response curves for numbers of M. hapla retrieved following treatment with Me. brunneum, strain ARSEF4556 or V275. Doses ranged from zero (control) to 10% (w/w). Nematodes were recovered from ‘Top’, ‘Middle’, and ‘Bottom’ sections of experimental tubes, 5 and 10 days, post treatment. Fitted lines show log-linear responses. Shaded ribbons show 95% confidence envelopes. A small amount of horizontal ‘jitter’ was applied to visually separate overlapping data points.

The total number of *M. hapla* J2s recovered post treatment from soils containing 1, 5 and 10% (w/v) *Me. brunneum* conidia declined with increasing dose for ARSEF4556 and V275 at the bottom of the tubes, after both 5 and 10 days post treatment (Fig. 5 bottom row panels). Very little effect of dose was seen in the middle section of the tubes (Fig. 5 middle row panels). At the top of the tubes, numbers declined with dose of ARSEF4556 after 5 days, but were consistently low across the range of doses after 10 days. With V275, numbers increased slightly with increasing dose at the top of the tubes; more so after 5 days than 10 days post treatment (Fig. 5 top row panels). Analysis of variance of pairwise interactions between ‘dose’ (0, 1, 5, 10%), ‘day’ (5, 10), ‘tube’ (top, middle, bottom), and ‘strain’ (ARSEF4556, V275) is summarised in Table 1. This analysis confirms that the only statistically significant pairwise interactions between explanatory variables were between dose and tube (p < 0.001), dose and strain (p = 0.004), and day and tube (p = 0.002).

**Table 1:** Analysis of variance of pairwise interactions between ‘dose’ (0, 1, 5, 10%), ‘day’ (5, 10), ‘tube’ (top, middle, bottom), and ‘strain’ (ARSEF4556, V275) explaining retrieval of *M. hapla* following treatment. All main effects are included in at least one statistically significant interaction so main effects do not need to be assessed individually.

**Interaction**	**F-ratio_df_**	**p-value**
dose × day	F_1,274_ = 1.393	p = 0.239
dose × tube	F_2,275_ = 12.31	p < 0.001
dose × strain	F_1,274_ = 8.539	p = 0.004
day × tube	F_2,275_ = 6.166	p = 0.002
day × strain	F_1,274_ = 0.331	p = 0.566
tube × strain	F_2,275_ = 0.677	p = 0.509

### 3.2 Behaviour of *M. hapla to Me. brunneum* conidia in choice study

**Fig. 6:**
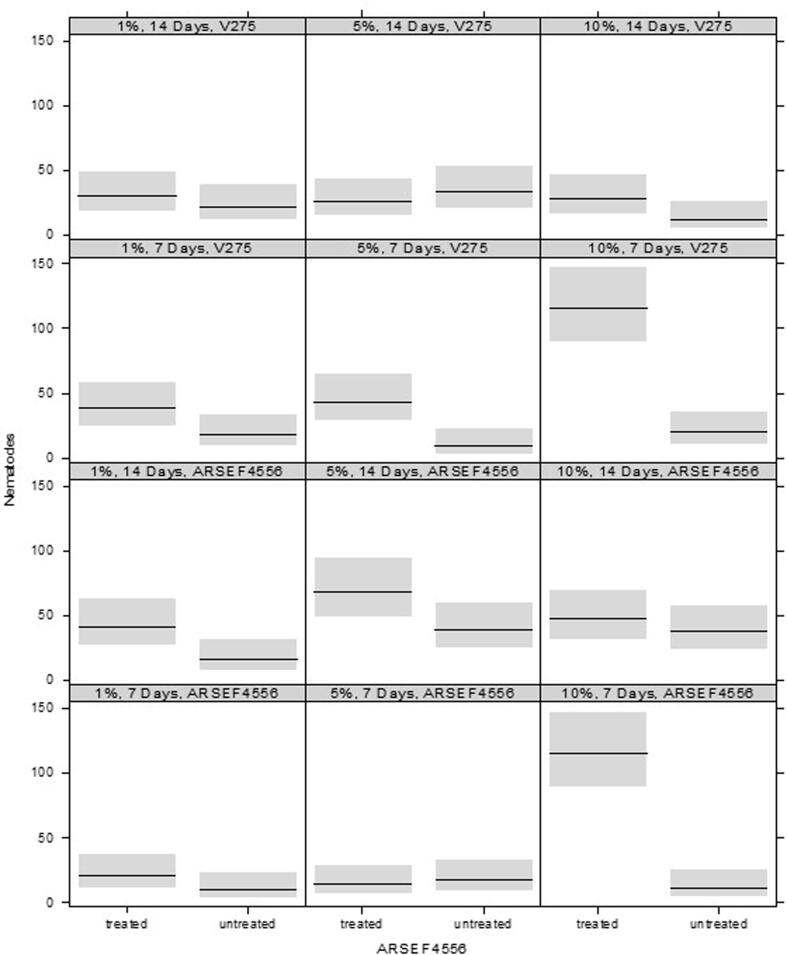
Numbers of nematodes attracted to either plants treated with Metarhizium or untreated plants. Midlines show median responses, with boxes spanning the interquartile ran.

Using *Metarhizium* strain ARSEF 4556, the three-way interaction between ‘dose’, ‘day’, and ‘treatment’ was statistically significant (F_2,62_ = 8.33, p < 0.001). Overall, more nematodes were attracted to treated than untreated plants. However, the degree of attraction was dependent on dose and days post treatment (Fig. 6, bottom two rows of panels). A similar result was observed with *Metarhizium* strain V275 (Fig. 6, top two rows of panels), with a dose-dependent increase in nematodes attracted to treated plants compared to untreated, but only seven days after treatment (treatment × days interaction: F_1,63_ = 10.5, p = 0.002).

### 3.3 Petri dish VOC fumigation assay against *M. hapla*

The VOCs 1-octen-3-ol (Fig. 7a) and 3-octanone (Fig. 7b) caused J2 mortality from 3 hrs post exposure at all the doses tested. Mortality increased with dose, time, and proximity to the source (1-octen-3-ol: dose × time F_2,636_ = 30.6, p < 0.001, dose × position F_2,636_ = 20.8, p < 0.001, time × position F_4,638_ = 2.45, p = 0.045; 3-octanone: dose × time × position F_4,634_ = 3.90, p = 0.004). 3-octanone appeared to be more toxic and covered a wider area than 1-octen-3-ol; for example, compare the transition from the Centre to Middle section of the arena, 3 h after treatment, for 3-octanone vs. 1-octen-3-ol (Fig. 7a & b). Closest to the VOC source, over 97% mortality was achieved for both compounds at the highest dose from 6 hrs post treatment. Highest mortalities were recorded 24 hrs post treatment for both compounds.

**Fig. 7a:**
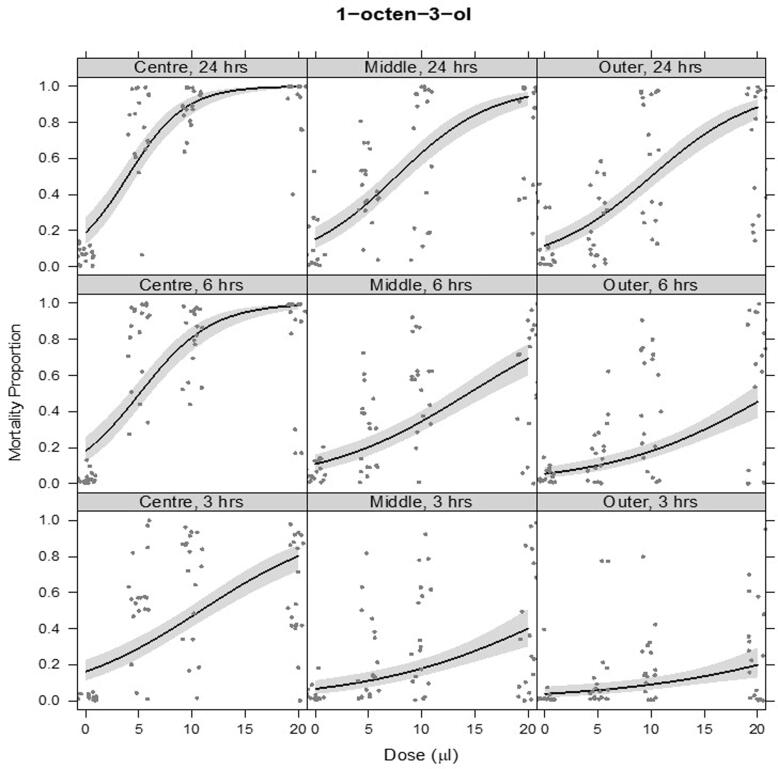
– 1-octen-3-ol. Proportional mortality of PPNs following VOC treatment over a range of dose concentrations (x-axis) and after varying times post treatment (panels by row: 3 hrs, 6 hrs, 24 hrs). This was assessed at varying distances from the centre of a circular arena (panels by column: Centre, Middle, Outer). Fitted lines are from a quasibinomial model of alive and dead nematodes. Shaded ribbons show 95% confidence envelopes. A small amount of horizontal ‘jitter’ was applied to visually separate overlapping data points.ge.

**Fig. 7b:**
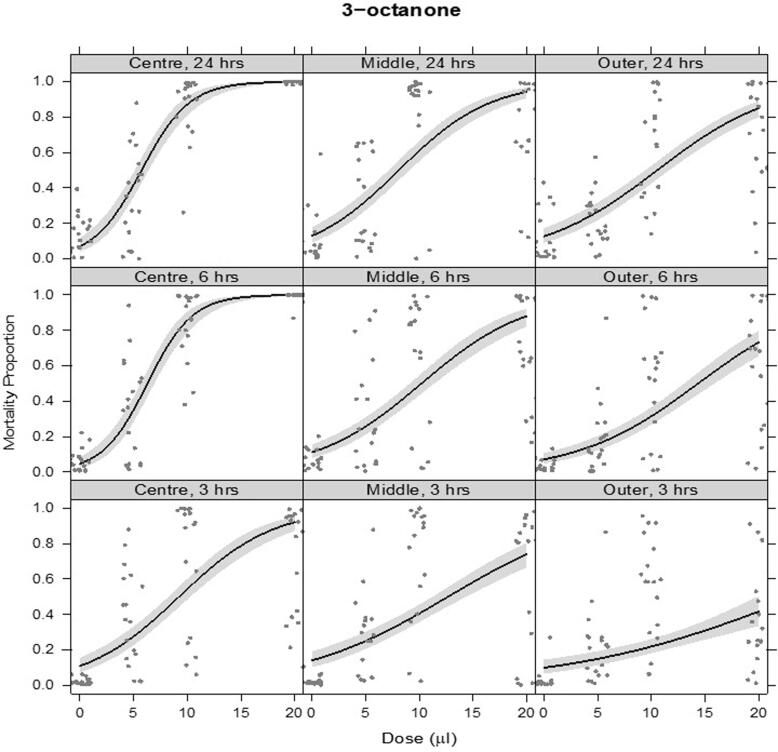
3-octanone. Proportional mortality of PPNs following VOC treatment over a range of dose concentrations (x-axis) and after varying times post treatment (panels by row: 3 hrs, 6 hrs, 24 hrs). This was assessed at varying distances from the centre of a circular arena (panels by column: Centre, Middle, Outer). Fitted lines are from a quasibinomial model of alive and dead nematodes. Shaded ribbons show 95% confidence envelopes. A small amount of horizontal ‘jitter’ was applied to visually separate overlapping data points.

### 3.4 Efficacy of formulated VOC against J2 *M. hapla* in soil

**Fig. 8:**
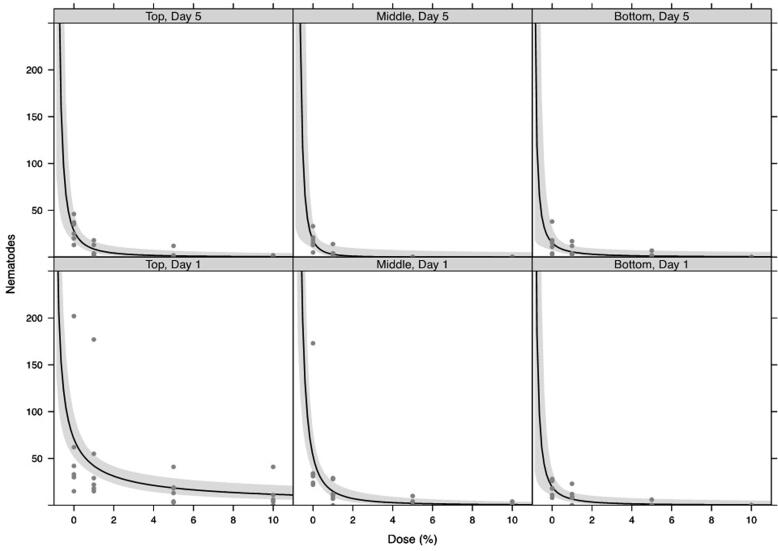
Dose-response curves for numbers of *M. hapla* retrieved following treatment with VOC 1-octen-3-ol. Nematodes were recovered from ‘Top’, ‘Middle’, and ‘Bottom’ sections of experimental tubes, after both 1 and 5 days post treatment. Fitted lines show log-linear responses. Shaded ribbons show 95% confidence envelopes.

Recovery of *M. hapla* J2s decreased with increasing dose of 1-octen-3-ol (Fig. 8). Recovery was generally lowest from the middle and bottom layers for both the 5% and 10% doses (dose × tube F_2,136_ = 4.30, p = 0.015). The number of nematodes recovered declined with time independent of dose (dose × day F_1,135_ = 3.39, p = 0.068).

### 3.5 Response of *M. hapla* to VOCs in choice studies

**Fig. 9:**
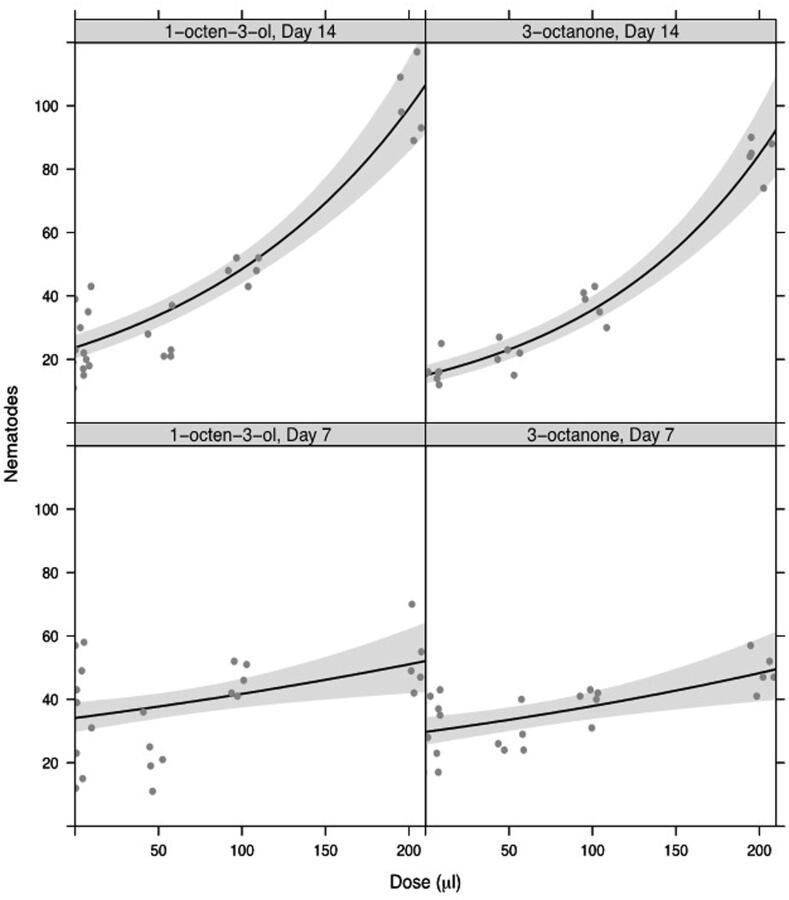
Dose-response curves for behavioural responses of *M. hapla* following treatment with VOCs, and assessed 7 and 14 days post treatment. Fitted lines show log-linear responses. Shaded ribbons show 95% confidence envelopes.

In choice studies where plants were exposed to both VOCs dispensed from filters, more *M. hapla* J2s were recovered from plants treated with 100 and 200 μl of 1-octen-3-ol and 3-octanone than 50 μl dose or untreated controls (Fig. 9). The numbers recovered increased with time with more being recovered after 14 days than 7 days post treatment, especially at the highest dose tested. Overall, there was a statistically significant interaction between dose (0, 1, 5, 10%) and day (7, 14) (F_1,114_ = 75.0, p < 0.001).

**Fig. 10:**
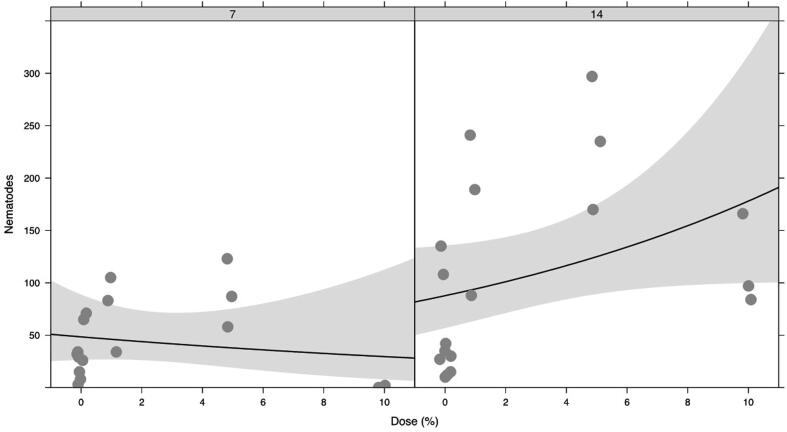
Dose-response curves for behavioural responses of M. hapla following treatment with VOC (granular formulation of 1-octen-3-ol) and assessed 7 and, 14 days post treatment. Fitted lines show log-linear responses. Shaded ribbons show 95% confidence envelopes.

In choice studies, there was no statistically significant interaction between dose (0, 1, 5, 10%) and day (7, 14) (dose × day F_1,33_ = 2.06, p = 0.161) nor a statistically significant effect of dose (F_1,34_ = 1.43, p = 0.241) (Fig. 10). However significantly more nematodes were present on day 14 (F_1,34_ = 10.4, p = 0.003) (Fig. 10).

### 3.6 Effect of VOCs on *M. hapla* behaviour in the presence of host plant (No choice study)

Nematode numbers were dose-dependent, with 1% of 1-octen-3ol or 3-octanone resulting in no greater numbers than controls whereas 5% of either compound resulting in 1.7 times greater numbers (Compound × dose: F_1,26_ = 0.465, p = 0.501; Compound: F_1,27_ = 0.027, p = 0.87; Dose: F_1,27_ = 18.1, p < 0.001).

## 4 Discussion

This is the first study to show that *Me. brunneum,* influences the behaviour of *M. hapla.* Conidia of this fungus appear to emit VOCs which attract the free living J2 nematodes, resulting in fewer *M. hapla* being extracted using the wet paper towel method. The recovery appears to be dependent on the fungal strain and dose, reflecting differences in the quantity and type of VOC emitted by each strain. EPF VOC profiles do differ between species, strains and cultural conditions (Bojke et al., 2018, Hussain et al., 2010, Mburu et al., 2013). The fact that PPN recovery was low from soils with high doses of conidia than untreated control suggests that *Me. brunneum* conidial volatiles were either powerful attractants or they had immobilised the nematodes in some way. The attractiveness was clearly dependent on the concentration of the VOCs since application of low doses of conidia had little effect. Hanel (1994), noted that PPN numbers increased after application of the EPF *Beauveria bassiana* (Bals.-Criv.) Vuill. to the soil due to indirect effects, including fungal chitinolytic activity which accelerated hatching. Similarly, Mwaura, et al., (Mwaura et al., 2017) found that application of *B. bassiana* resulted in a rise in the population of the PPN, *Ditylenchus destructor* Thorne and *Ditylenchus dipsaci* Kuhn, with corresponding increased damage to potato tubers. However, these workers failed to explain how the fungus lured these nematodes. Other workers have reported a decline in PPN numbers. For example, drenching of soil with spores or fermented products of *B. bassiana* resulted in a decline in *M. hapla* numbers with a concomitant reduction in plant damage (Liu et al., 2008). However, not all *B. bassiana* strains secrete nematicidal compounds (Zhao et al., 2013). These observations, and those of the current study, suggest that careful attention needs to be given to the EPF strain and application rates since deployment of EPF in insect pest management programmes may inadvertently attract PPN and increase crop losses.

Although there is much information on EPF VOCs influencing insect behaviour (Butt et al., 2016). little is known about their effect on other soil invertebrates. The current study shows that *Me. brunneum* VOCs, more specifically 1-octen-3-ol and 3-octanone, are attractive at low concentrations but toxic at relatively higher doses. Direct exposure of PPN to these volatiles results in death with the speed of death dependent on dose and time. Several soil fungi and bacteria are known to produce nematicidal VOCs which reduce hatching and infectivity including: *Fusarium oxysporum* Schlecht. emend. Snyder & Hansen strain 21 (Terra et al., 2018); *Trichoderm*a sp Persoon (Yang et al., 2019); and several strains of bacteria belonging to the genera *Bacillus* sp. Cohn, *Paenibacillu*s sp Ash *et al*., and *Xanthomonas* sp Dowson (Bui, et al., 2019). The fact that each microbe emits nematicidal VOCs with different chemistries suggests these traits evolved independently, presumably to protect the host plant (source of refuge and food) from detrimental PPN or to escape predation by fungus and bacterium eating nematodes (Yang et al., 2019, Haraguchi and Yoshiga, 2020).

Evaluation of prototype formulations of 1-octen-3-ol and 3-octanone show that they have potential for use as soil fumigants. However, based on the current loadings, application rates would have to be optimised for different soil types. It is possible that higher (i.e. > 10% w/w) application rates may be required, which may be costly for growers. The ability of low doses of the VOCs to lure *M. hapla* to host plant roots suggests that they have the potential for luring the pest to trap crops allowing for targeted control of this pest. This strategy would reduce application rates of biological and chemical pesticides. The low dose VOCs could also be used as monitoring tools to identify hot spots in the field or determine the efficacy of control agents. Higher loadings and improved controlled release of the actives could mean the VOCs could be used as repellents, giving the crop the chance to grow and develop natural immunity to the pest. The future of 1-octen-3-ol and 3-octanone as plant protection products is promising not only because of the number of ways these compounds can be used but also because they possess other attributes sought by growers including: leaving no residues and repelling other crop pests such as slugs and snails (Khoja et al., 2019).

## 5 Conclusions

Conidia of *Me. brunneum* conidia produce VOCs which attract the RKN, *M. hapla*. The VOCs, 1-octen-3-ol and 3-octanone, attract free living J2 *M. hapla* at low concentrations and repel or kill them at high concentrations.

## CRediT authorship contribution statement

**Salim Khoja:** Conceptualization, Investigation, Methodology, Formal analysis, Writing - review & editing. **Khalifa M. Eltayef:** Investigation, Methodology, Formal analysis, Writing - review & editing. **Ian Baxter:** Conceptualization, Funding acquisition, Resources, Methodology, Writing - review & editing. **Arben Myrta:** Conceptualization, Resources, Methodology, Writing - review & editing. **James C. Bull:** Formal analysis, Writing - review & editing. **Tariq Butt:** Conceptualization, Funding acquisition, Resources, Investigation, Methodology, Writing - original draft, Writing - review & editing.

## Declaration of Competing Interest

The authors declare that they have no known competing financial interests or personal relationships that could have appeared to influence the work reported in this paper.
